# Microglia Polarization from M1 toward M2 Phenotype Is Promoted by *Astragalus Polysaccharides* Mediated through Inhibition of miR-155 in Experimental Autoimmune Encephalomyelitis

**DOI:** 10.1155/2021/5753452

**Published:** 2021-12-24

**Authors:** Xiaojun Liu, Jinyun Ma, Guiqing Ding, Qianyi Gong, Yuanhua Wang, Hua Yu, Xiaodong Cheng

**Affiliations:** Institute of Clinical Immunology, Yue-Yang Hospital of Integrative Medicine, Shanghai University of Traditional Chinese Medicine, Shanghai 200437, China

## Abstract

Activated microglia is considered to be major mediators of the neuroinflammatory environment in demyelinating diseases of the central nervous system (CNS). Activated microglia are mainly polarized into M1 type, which plays a role in promoting inflammation and demyelinating. However, the proportion of microglia polarized into M2 type is relatively low, which cannot fully play the role of anti-inflammatory and resistance to demyelinating. Our previous study found that *Astragalus polysaccharide*s (APS) has an immunomodulatory effect and can inhibit neuroinflammation and demyelination in experimental autoimmune encephalomyelitis (EAE), which is a classic animal model of CNS demyelinating disease. In this study, we found that APS was effective in treating EAE mice. It restored microglia balance by inhibiting the polarization of microglia to M1-like phenotype and promoting the polarization of microglia to M2-like phenotype *in vivo* and in *vitro*. miR-155 is a key factor in regulating microglia polarization. We found that APS could inhibit the expression level of miR-155 *in vivo* and *in vitro*. Furthermore, we performed transfection overexpression and blocking experiments. The results showed that miR-155 mediated the polarization of microglia M1/M2 phenotype, while the selective inhibitor of miR-155 attenuated the inhibition of APS on microglia M1 phenotype and eliminated the promotion of APS on microglia M2 phenotype. Microglia can secrete IL-1*α*, TNF-*α*, and C1q after polarizing into M1 type and induce the activation of A1 neurotoxic astrocytes, further aggravating neuroinflammation and demyelination. APS reduced the secretion of IL-1*α*, TNF-*α*, and C1q by activated microglia, thus inhibited the formation of A1 neurotoxic astrocytes. In summary, our study suggests that APS regulates the polarization of microglia from M1 to M2 phenotype by inhibiting the miR-155, reduces the secretion of inflammatory factors, and inhibits the activation of neurotoxic astrocytes, thus effectively treating EAE.

## 1. Introduction

Over the past decades, there has been growing evidence regarding the importance of herbs in traditional Chinese medicine (TCM) in the treatment of demyelination disease of the central nervous system (CNS), which classic animal model is experimental autoimmune encephalomyelitis (EAE) [1]. *Astragalus polysaccharides* (APS) is one of the key active components of *Astragalus membranaceus*. Pharmacological experiments have shown that APS has multiple pharmacological effects, such as anti-inflammatory, antitumor, and immune regulation [2–5]. Our previous study found that APS could significantly inhibit neuroinflammation and demyelination in EAE mice [6]. But the mechanism of action is not fully understood.

It is widely accepted that microglia are tissue macrophage-like innate immune cells that reside in the CNS [7], which are considered as key immune monitors and responders in the CNS and play a pivotal role in the pathogenesis of demyelination disease [8]. Microglia plays a highly active monitoring role in the physiological state through various surface receptors, which can be activated by sensing subtle changes in their microenvironment and responding to these changes. Activated microglia undergo a dramatic morphological and functional transformation, with proinflammatory and anti-inflammatory cytokines releasing [9]. During the early stages of the disease, the M1-like phenotype highly upregulates that many classical inflammatory mediators, like tumor necrosis factor-alpha (TNF-*α*), induced nitric oxide synthase (iNOS), and interleukin-12 (IL-12), showed to be destructive to the myelin sheath. In contrast, during the later stages of the disease, the M2-like phenotype notably upregulates anti-inflammatory mediators such as Arginase-1 (Arg-1), interleukin-10 (IL-10), and CD206, which are displayed to be beneficial to myelin sheath [10–12]. Hence, developing new drugs in promoting the polarization of microglia from the proinflammatory phenotype to the anti-inflammatory phenotype may be an effective treatment strategy in EAE.

The miRNAs, which are noncoding RNA sequences, are the master regulators of the target messenger-RNA (mRNA), which are involved in inhibiting the transcriptional level of target genes or directly degrade target genes [13]. At present, it has been found that miRNA-155 can promote the release of inflammatory cytokines and participate in the immune response process [14, 15]. The expression of miR-155 in peripheral blood mononuclear cells of patients with relapsing-remitting multiple sclerosis is significantly upregulated [16], indicating that miR-155 is involved in the progression of multiple sclerosis. Additionally, significantly over expression of miR-155 in the lumbar spinal cord of EAE mice was demonstrated [17]. Besides, the expression of miR-155 in splenic CD4 + T lymphocytes of EAE mice was significantly increased, and systematic knockout of miRNA-155 could reduce the ratio of Th1 to Th17 in central and peripheral inflammatory cells of EAE mice, thus reducing the severity of the disease [18]. Furthermore, a previous study demonstrated that miR-155 is vital for the polarization of microglia to the M1 phenotype, while the absence of miR-155 causes microglia to the M2 phenotype polarization [19], suggesting that miR-155 is involved in the occurrence and development of EAE and may be a new target for the therapy of EAE.

The activated microglia secreted inflammatory factors including TNF-*α*, IL-1*α*, and C1q to induce the formation of A1 astrocytes. All three factors were indispensable for the formation of A1 astrocytes, which have significant neurotoxicity and are also called A1 neurotoxic astrocytes. The increase of GFAP is a symbolic signal of astrocytes activation, and the complement component C3 is one of the most characteristic and highly upregulated genes in A1 astrocytes. Therefore, C3 and GFAP are currently used as marker molecules to identify the formation of A1 astrocytes. The neuroinflammatory environment of EAE promotes the activation of microglia, thus inducing the formation of a large number of A1 astrocytes, producing neurotoxin substances, leading to the death of oligodendrocytes and neurons, demyelination, and axon loss, and promoting the progression of the disease [20, 21]. Inhibiting the formation of A1 astrocytes and reducing their neurotoxicity can prevent the death of oligodendrocytes and neurons, inhibit demyelination and axon loss, and thus effectively treat EAE [22].

Here, we found that APS could inhibit the polarization of microglia to M1 type and promote towards the M2 type in EAE mice *in vivo* and in activated microglia *in vitro*. Based on it, we demonstrated that miR-155 may mediate the regulation of APS on the phenotype polarization of microglia. Further, APS inhibited the secretion of inflammatory factors that induce A1 astrocytes by activated microglia and then suppressed the activation of A1 astrocytes. This study highlighted the importance of the regulatory effect of APS on CNS inflammation and provides new targets and ideas for TCM treatment of neurodegenerative diseases in the CNS.

## 2. Materials and Methods

### 2.1. Animals

Healthy adult female C57BL/6J mice (6-8 weeks of age) were from JiHui Experimental Animal Breeding Co., Ltd. (Shanghai, China). Mice were housed in specific pathogen-free (SPF) conditions in a 12-hour dark circulation room with temperature and humidity controlled, with ad libitum access to food and water at the animal care facility of the Yue-yang Hospital of Integrative Medicine, Shanghai University of Traditional Chinese Medicine. All surgical procedures were carried out following protocols approved by the Institutional Animal Care at the Yue-yang Hospital of Integrative Medicine, Shanghai University of Traditional Chinese Medicine (permit number: 18902).

### 2.2. EAE Induction and Treatment

To induce of active experimental autoimmune encephalomyelitis (EAE), female mice aged 6-8 weeks C57BL/6 mice underwent subcutaneous immunity on day 0 with 200 mg MOG_35-55_ peptide (Genescript, NJ, USA) emulsified in complete Freund's adjuvant (CFA supplemented with 5 mg/ml Mycobacterium tuberculosis H37Ra) (Sigma-Aldrich, St. Louis, MO, USA), followed by intraperitoneal administration of 300 ng pertussis toxin (PTX) (Millipore, MA, USA) on days 0 and 2. Clinical signs of EAE were assessed based on the following score [23]: 0, no signs of disease; 1, loss of tone in the tail; 2, hind limb paresis; 3, complete hind limb paralysis; 4, tetraplegia; and 5, moribund. For the treatment, APS was given by oral administration 500 mg/kg since the day of immunization (day 0), and normal saline solution has been given as a vehicle control (200 *μ*l/d).

### 2.3. Reagents

Lipopolysaccharide (LPS) was purchased from Sigma-Aldrich (St. Louis, MO, USA); *Astragalus polysaccharides* (APS, UV ≥ 98%) were bought from Yuan-ye Biotechnology (Shanghai, China). RPMI-1640 medium, penicillin-streptomycin solution, 0.05% trypsin-digestion solution, and fetal bovine serum were obtained from Gibco (Grand Island, NY, USA). Phosphate buffer (PBS) was purchased from HyClone (South Logan, UT, USA). Rabbit anti-GAPDH, rabbit anti-iNOS, rabbit anti-Arg-1, mouse anti-IBA-1, anti-rabbit IgG HRP-linked antibody, and anti-mouse IgG HRP-linked antibody were obtained from Cell Signaling Technology (CST, Danvers, MA, USA). Goat anti-mouse IgG H + L antibody was obtained from Biotech (Beijing, China).

### 2.4. Cell Culture and Treatment

The BV-2 cell is an immortalized murine microglia line which was acquired from China Center for Type Culture Collection (Kunming, China). In 95% air and 5% CO2 atmosphere, cells were cultured in RPMI-1640 medium with 10% FBS and 1% penicillin-streptomycin at 37°C. Cells were seeded into 6-well plates at 2.5 × 10^5^ cells per cm^2^, 24-well plates at 2 × 10^3^ cells per cm^2^, or 96-well plates at 1 × 10^5^ cells per cm^2^. Cultures were preprocessing with APS (0.4 mg/ml) for 24 h, and the M1-polarized BV-2 cells were generated by a 24-hour treatment with LPS at 1 *μ*g/ml (L2880-10MG, St. Louis, MO, USA). The corresponding indexes were tested.

### 2.5. siRNA Gene Silencing and MicroRNA Transient Transfection

Overexpression or inhibition of miR-155 was achieved by delivery of anti-miR-155 oligonucleotides or plasmid DNA encoding miR-155, respectively, to BV-2 microglia cells. A total of 1 × 10^5^ cells per cm^2^ were separately mixed with Cy3-miR-ON/OFF NC (50 nmol/100 nmol, RIBORIO Co., Ltd., China), miR-155 mimics (50 nmol, RIBORIO Co., Ltd., China), miR-155 inhibitor (100 nmol, RIBORIO Co., Ltd., China), or their corresponding control (RIBORIO Co., Ltd., China) in riboFECT™ CP Reagent using riboFECT™ CP Transfection Kit (C10511-1, RIBORIO, China) at room temperature for 24 h. Then, cells were incubated with APS (0.4 mg/ml) for 24 h and another 24 h incubated with LPS (1 mg/ml) at 37°C; cells were collected for extracting total RNA or protein.

### 2.6. The Efficiency of miRNA-155 Fluorescence Transfection

The efficiency of miRNA-155 fluorescence transfection was measured 24 hours later, the cells expressing red fluorescence were counted by immunofluorescence inverted microscope, and the images were scanned and collected. Two to three high-power microscope visual fields were randomly selected to calculate the percentage of cells expressing red fluorescence in the total number of cells in each field (transfection rate = fluorescent expression cells/total cells × 100%), and the average value was taken as the transfection efficiency.

### 2.7. CCK8 Assay

Cells were seeded into 96-well plates at 1 × 10^5^ cells per cm^2^. 100 *μ*l CCK8 solution was added to each well and incubated at 37°C for 1-4 h. The absorbance was measured at 450 nm.

### 2.8. Immunocytochemical Staining

BV-2 cells were fixed at room temperature with 4% paraformaldehyde for 30 min followed by 0.3% Triton X-100 (Beyotime, Shanghai, China) permeability for 15 min. Cells were then blocked at room temperature for 40 min with 2% BSA (Biofroxx, Germany). Cells were concentrated overnight at 4°C with anti-Iba-1 (1: 200). The next day, cells were incubated with Alexa Fluor 488-conjugated anti-mouse-IgG (1: 200) antibody for 1 hour at room temperature. Subsequently, cells were incubated with DAPI (Solarbio, Beijing, China) for 10 min at room temperature.

### 2.9. RNA Isolation and qRT-PCR Assay

Total RNA was extracted from mice and BV-2 microglia cells according to a standard protocol applying TRIzol® reagent (Thermo Fisher, Waltham, MA, USA). Total RNA was reverse transcribed by using iScript cDNA Synthesis Kit (BIO-RAD, Hercules, CA, USA). iTaq Universal SYBR Green Supermix (SDS-PAGE electrophoresis) at 10 *μ*l volumes was used for real-time PCR reactions in 8-Tube Strips using the Thermo Fisher ABI QuantStudio 7 Flex (Thermo Fisher, Waltham, MA, USA). In this process, the primer-dimers and nonspecific amplification products were not identified. Standardize GAPDH as an endogenous control and the comparative threshold cycle method 2^-∆∆CT^ were used to calculate the relative expression. The results were expressed as fold changes relative to the control group.

To determine miRNA expression, TRIzol® reagent was used for extracting total RNA, including miRNA. A total of 1 *μ*g of total RNA was reverse transcribed using ribo Bulge-Loop™ miRNA qRT-PCR Starter Kit (RIBORIO Co., Ltd., China) with miRNA-specific primers. Reverse transcription reaction products (2 *μ*l) were used for real-time PCR as described above. The following ribo miRNA expression assays were used: miR-155-5p and endogenous control U6 small nuclear RNA (snRNA). Reactions were amplified and quantified using the Thermo Fisher ABI QuantStudio 7 Flex. U6 small nuclear RNA was used as an endogenous control for data normalization, and the comparative threshold cycle method 2-^∆∆CT^ was used to calculate the relative expression. The primers for gene amplification were as Table 1.

### 2.10. Western Blot Analysis

It was used to detect the expression of microglia activated marker IBA1, M1 phenotypic marker iNOS protein, and M2 phenotypic marker Arg-1 protein. Briefly, microglia were lysed in RIPA strong lysis buffer on ice and then centrifuged at 4°C and 12000 rpm for 10 min to extract nucleoprotein, and quantitative analysis of protein content was performed by BCA (Beyotime, Shanghai, China) method. The protein components of the sample were separated on SDS-PAGE gels (BIO-RAD, Hercules, CA, USA) and transferred to the PVDF membrane (Biosharp, Suzhou, China). After blocking with 5% skimmed milk (BIO-RAD, Hercules, CA, USA), the membranes were incubated with the primary antibodies against GAPDH, IBA1, iNOS, and Arg-1 overnight at 4°C. After the primary antibody incubation is completed, the PVDF membranes were incubated with HRP conjugated IgG antibodies for 1 h at room temperature and analyzed by an image with a Chemiscope imaging system (CLiNX, Shanghai, China). The intensities of target protein bands were quantified and normalized by GAPDH using ImageJ (NIH, Bethesda, MD).

### 2.11. Statistical Analysis

Data were presented as mean ± standard deviation of the mean (SD). Statistical significance was analyzed by unpaired two-tailed Student's *t*-test or one-way analysis of variance (ANOVA) through the GraphPad Prism software (GraphPad Software Inc., San Diego, CA, USA). *P* < 0.05 indicates that the difference is statistically significant.

## 3. Results

### 3.1. APS Alleviated Neurological Dysfunction in EAE Mice

**Figure 1:**
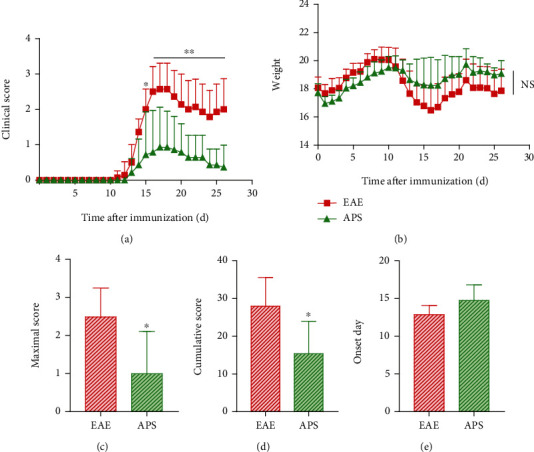
APS alleviated neurological dysfunction in EAE mice. Clinical score (a), body weight (b), maximal score (c), cumulative score (d), and onset day (e) of the EAE and APS group were measured daily from day 0 to day 26 (*n* = 7). Compared with the EAE group, ^∗^*P* < 0.05, ^∗∗^*P* < 0.01.

To clarify the therapeutic effect of APS on EAE mice, the clinical score and body weight of each group were dynamically monitored daily during the whole experiment. The mice in the EAE group started to show neurological deficit symptoms on the 11th day; the peak time occurred on the 16th day, and the clinical score of EAE mice increased sharply. The remission period of EAE mice began on the 19th day. From the 16th day after modeling, the clinical score in the APS group was significantly lower than that in the EAE group (Figure 1(a)), and the maximal score and the cumulative score in the APS group were also noticeably reduced (Figures 1(c) and 1(d)). After MOG_35-55_ induced the EAE model, the body weight of the mice in the EAE group and the APS group decreased and gradually recovered on the third day after immunization. From the 11th day after immunization, the body weight of the EAE mice decreased remarkably, but there is no significant difference between the EAE group and APS group of the body weight and the onset day (Figures 1(b) and 1(e)).

### 3.2. APS Inhibited the Activation of Microglia In Vitro

**Figure 2:**
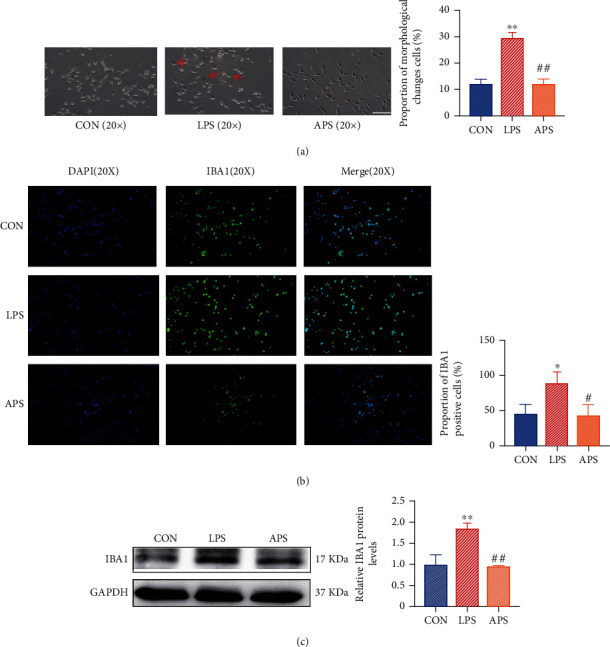
APS inhibited LPS-induced microglia activation. Morphological changes of microglia in each group (a). The expression of the IBA1 protein was detected by immunofluorescence staining and western blot in each group (b, c). Compared with the CON group, ^∗^*P* < 0.05, ^∗∗^*P* < 0.01. Compared with the EAE group, #*P* < 0.05, ##*P* < 0.01.

To verify the effect of APS on microglia, firstly, we certified the effect of APS on LPS-induced activated microglia (BV-2 cell line) *in vitro*. The CCK8 assay showed that APS (0.8 mg/ml) remarkably inhibited the cell viability of BV-2 cells; APS (0.4 mg/ml) and LPS (1 *μ*g/ml) had no significant effects on the cell viability of BV-2 cells (Fig. S1); 0.4 mg/ml was selected as the concentration of APS in the follow-up study. The morphology of microglia in the CON group showed small fusiform bodies with thin and long pseudopodia; in the LPS-stimulated group, activated microglia exhibited round bodies with shrank and shortened pseudopodia, even showed amoeba-like changes; in the APS pretreated group, part of microglia manifested a similar morphology to the CON group (Figure 2(a)). It is found that APS could reshape the morphological changes of microglia induced by LPS. Furthermore, immunofluorescence and western bolt methods were used to detect the expression level of IBA1, a marker of microglia activation [24] (Figures 2(b) and 2(c)). APS could inhibit the increasing of IBA-1 protein expression induced by LPS. These data indicated that APS could effectively inhibit the activation of microglia *in vitro*. Furthermore, microglia can polarize into M1 type or M2 type after activation. Whether APS inhibited the polarization of both phenotypes or mainly inhibited one phenotype. In the next experiment, we observed the effect of APS on M1 and M2 polarization phenotype microglia, respectively.

### 3.3. APS Promoted the Phenotypic Polarization of Microglia from M1 to M2 In Vitro

**Figure 3:**
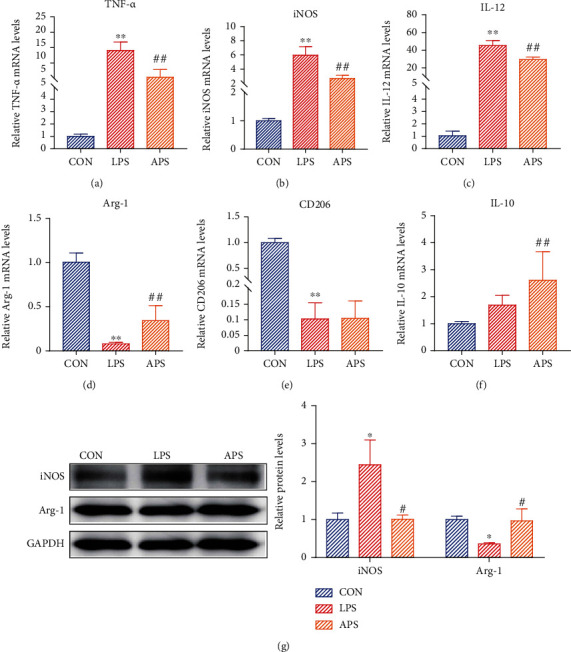
APS promoted the phenotypic polarization from M1 to M2 of microglia *in vitro*. Relative mRNA expression levels of M1 polarization markers (TNF-*α*, iNOS, IL-12) and M2 polarization markers (Arg-1, IL-10, CD206) were detected by qRT-PCR (a–f). The protein expression of M1 polarization marker (iNOS) and M2 polarization marker (Arg-1) was detected by western blot (g). Compared with the CON group, ^∗^*P* < 0.05, ^∗∗^*P* < 0.01. Compared with the EAE group, #*P* < 0.05, ##*P* < 0.01.

It is widely accepted that TNF-*α*, iNOS, and IL-12 were utilized as the marker of M1 phenotype, whereas Arg-1, IL-10, and CD206 were served as the marker of M2 phenotype. To discern the effects of APS on the polarization of microglia, the expression of M1 and M2 polarization markers was detected in each group, respectively. Compared with the CON group, the mRNA expression of the M1 maker TNF-*α*, iNOS, and IL-12 was appreciably enhanced (Figures 3(a)–3(c)), while the M2 markers Arg-1 and CD206 were decreased in the LPS group (Figures 3(d)–3(f)). Surprisingly, the differences in IL-10 expression were not obvious (Figure 3(f)). The APS-treated group expressed a lower level of proinflammatory molecule TNF-*α*, iNOS, and IL-12 (Figures 3(a)–3(c)). As expected, a higher level of anti-inflammatory molecule Arg-1 and IL-10 in the APS group than in the LPS group (Figures 3(d)–3(f)). Interestingly, the expression of CD206 was not significantly difference between the LPS group and the APS group (Figure 3(e)). Besides, in line with mRNA expression, the expression of iNOS protein of M1 type marker molecule was increased, and the expression of Arg-1 protein surface of M2 type marker molecule was decreased in the LPS group. APS completely reversed this state, the expression of iNOS protein decreased, and the expression of Arg-1 protein increased in the APS group (Figure 3(g)). Collectively, our data showed that APS inhibited M1 polarization and promoted M2 polarization of microglia *in vitro*.

### 3.4. APS Promoted the Phenotypic Polarization of Microglia from M1 to M2 in EAE Mice

**Figure 4:**
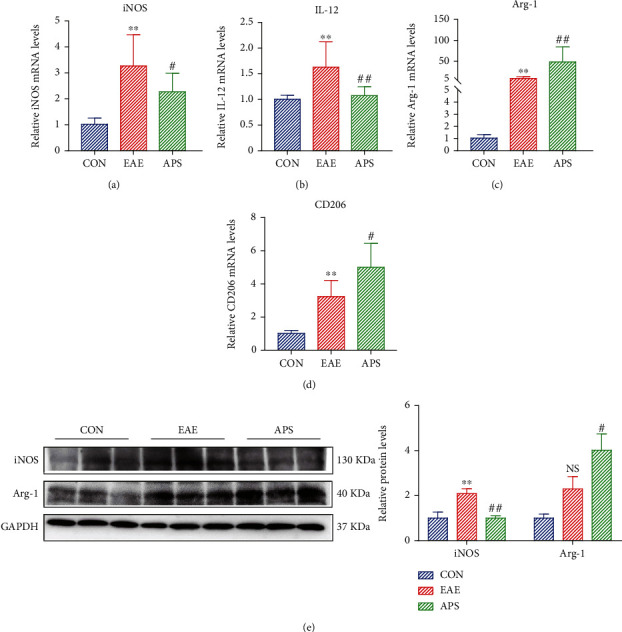
APS promoted the phenotypic polarization from M1 to M2 of microglia in EAE mice. Relative mRNA expression levels of M1 polarization markers (iNOS, IL-12) and M2 polarization markers (Arg-1, CD206) were detected by qRT-PCR (a–d). The protein expression of iNOS and Arg-1 was detected by western blot (e). Compared with the CON group, ^∗^*P* < 0.05, ^∗∗^*P* < 0.01. Compared with the EAE group, #*P* < 0.05, ##*P* < 0.01.

During EAE, M1-like microglia exerted a destructive impact on the myelin sheath, whereas M2-like microglia played a protective effect on myelin. In the previous results, APS could promote that the transformation of microglia from M1 to M2 was confirmed *in vitro*. Furthermore, we explored whether APS also has the same regulative effect in EAE mice. The expression of M1 polarization markers (iNOS, IL-12) and M2 polarization markers (Arg-1, CD206) was detected in the brain of EAE mice by qRT-PCR. Analyses showed that the expression of M1 type markers iNOS and IL-12 mRNA in the EAE group was significantly increased compared to the CON group (Figures 4(a) and 4(b)), while that in the APS group was notably decreased. In the meantime, the expression of M2 type markers Arg-1 and CD206 mRNA were increased in the EAE group, while it was further increased in the APS group, which was significantly higher than the other two groups (Figures 4(c) and 4(d)). Consistent with mRNA expression levels, protein levels of M1 phenotypic marker (iNOS) and M2 phenotypic marker (Arg-1) were increased in the EAE group. In the APS group, iNOS decreased significantly, and Arg-1 increased further (Figure 4(e)). These data indicated that APS could effectively suppress the polarization of microglia to M1 phenotype and promote the polarization of microglia to M2 phenotype in EAE mice.

### 3.5. APS Inhibited the Expression of miR-155 in Microglia

**Figure 5:**
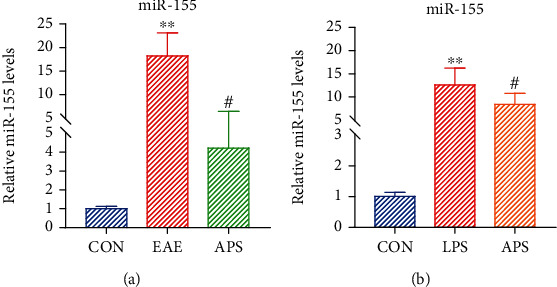
APS inhibited the expression of miR-155 in microglia. Relative mRNA expression levels of miR-155 were detected by qRT-PCR (a, b). Compared with the CON group, ^∗∗^*P* < 0.01. Compared with the EAE/LPS group, #*P* < 0.05.

Several studies have demonstrated that miR-155 plays proinflammatory effects in demyelination diseases. The downregulation of miR-155 plays a neuroprotective role. However, it remains unknown whether APS can suppress the expression of miR-155. In this study, we observed the expression of miR-155 in the brain tissue of EAE mice and activated microglia by LPS induced. Results showed that the expression of miR-155 in the brain of EAE mice was strikingly increased; by contrast, it was sharply decreased after APS treatment (Figure 5(a)). *In vitro*, under the induction of LPS, the expression of miR-155 in BV-2 cells was significantly upregulated, while after pretreatment with APS, it was steeply downregulated (Figure 5(b)). It is suggested that APS could inhibit the expression of miR-155 *in vivo* and *in vitro*.

### 3.6. miR155-Mediated APS Regulated the Phenotypic Polarization of Microglia from M1 to M2

**Figure 6:**
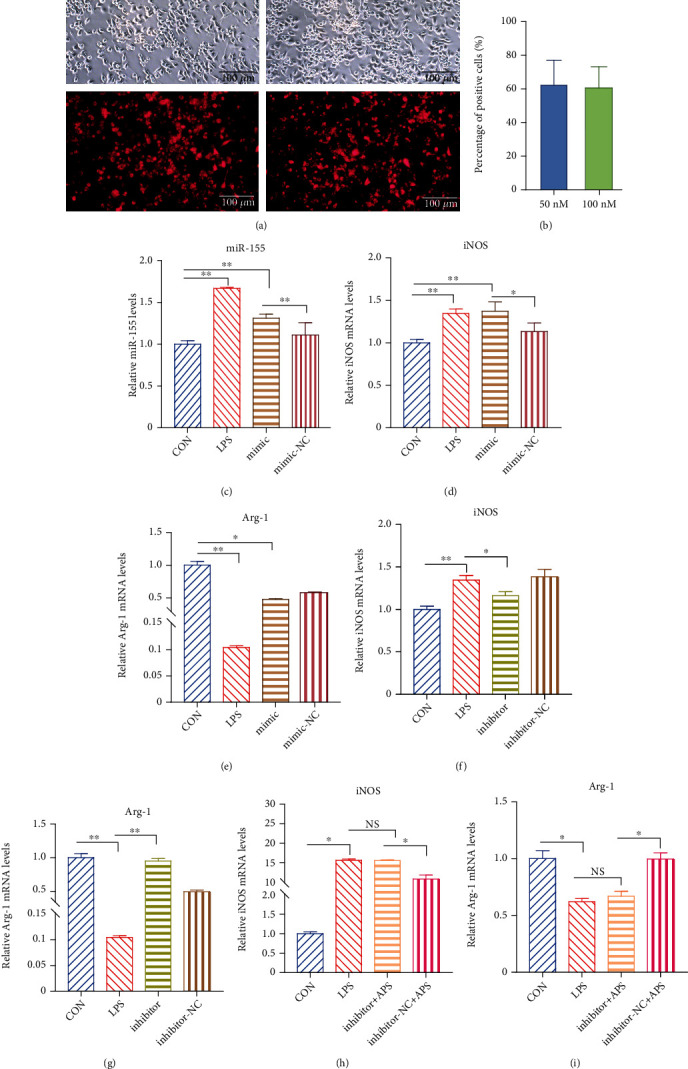
miR-155-mediated APS regulated the phenotypic polarization of microglia. The transfection efficiency was observed by fluorescence imaging (a, b). The expression level of miR-155 was detected by qRT-PCR (c). The relative mRNA expression levels of M1 polarization markers (iNOS) and M2 polarization markers (Arg-1) were detected under the induction of miR-155 mimic (d, e) and inhibitor (f, g) by QRT-PCR. The relative mRNA expression levels of iNOS and Arg-1 after APS intervention induced by miR-155 inhibitor (h, i) by QRT-PCR. ^∗^*P* < 0.05, ^∗∗^*P* < 0.01.

The bulk of the research has shown that miR-155 is considered to be a crucial regulator gene in the polarization of microglia. We further confirmed that the effect of miR-155 is related to the polarization of microglia via cell transfection. Obvious red fluorescence appeared in the cytoplasm of microglia transfected with 50 nM and 100 nM Cy3 miR-155 NC, and the fluorescence efficiency of transfection could reach more than 60% (Figures 6(a) and 6(b)). Real-time fluorescent quantitative PCR results showed that miR-155 mimic could significantly upregulate the expression of miR-155 compared with the CON group and miR-155 mimic NC group (Figure 6(c)). Compared with the CON group, miR-155 mimic can significantly upregulate the mRNA expression of M1 marker iNOS (Figure 6(d)) and downregulate the mRNA expression of M2 marker Arg-1 (Figure 6(e)). The transfection of the miR-155 inhibitor could drastically reduce the mRNA expression of iNOS (Figure 6(f)) and increase the mRNA expression of Arg-1 (Figure 6(g)). It showed that miR-155 partially mediated the transformation of the M1/M2 polarization phenotype of microglia.

Studies have shown that the expression of miR-155 increased in microglia and macrophages in EAE, causing persistent neuroinflammation. Meanwhile, inhibiting the expression of miR-155 can reduce neuroinflammatory responses [25]. APS could promote the transformation of microglia from M1 to M2 polarized phenotype. At the same time, APS could inhibit the expression of miR-155 in microglia induced by LPS. However, whether APS regulated the polarization of microglia phenotype by inhibiting the expression of miR-155 is vague. Therefore, we selected miRNA-155 inhibitors to verify whether APS regulated the M1/M2 polarization phenotype of microglia through miR-155. The expression of miR-155 was significantly upregulated under the induction of miR-155 mimic and LPS. miR-155 mimic and LPS could induce microglia to polarize to the M1 phenotype, while APS could inhibit its polarization to the M1 phenotype. Compared with miR-155 inhibitor NC, under the action of miR-155 inhibitor, the inhibiting effect of APS on the M1 polarization phenotype was weakened (Figure 6(h)). Besides, we further found that compared with miR-155 inhibitor NC, miR-155 inhibitor partially abolished the effect of APS in promoting M2 polarization (Figure 6(i)). Therefore, the above results indicated that APS could reverse the upregulation of M1 type markers and the downregulation of M2 type markers induced by LPS. The effect of APS is mainly partly dependent on the pathway of inhibiting miR-155 to polarize microglia from M1 phenotype converted to M2 polarized phenotype.

### 3.7. APS Inhibited Inflammatory Factors That Induce A1 Astrocyte Formation by Microglia and the Formation of A1 Astrocytes in EAE Mice

**Figure 7:**
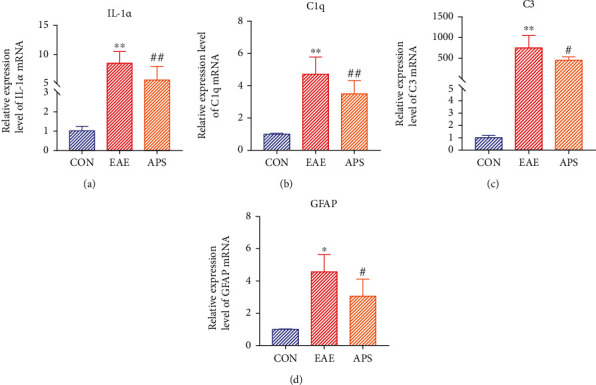
APS inhibited the mRNA level of IL-1*α* and C1q that induce activation of A1 astrocytes and in EAE mouse brain tissue (a, b) and suppressed the mRNA level of A1 astrocyte marker molecules C3 and GFAP (c, d) by qRT-PCR. Compared with the CON group, ^∗^*P* < 0.05, ^∗∗^*P* < 0.01; compared with the EAE group, #*P* < 0.05, ##*P* < 0.01.

The activated microglia secreted inflammatory factors including TNF-*α*, IL-1*α*, and C1q to induce the formation of A1 astrocytes. In the part of 3.3, we have demonstrated that APS decreased the expression level of TNF-*α* mRNA secreted by activated microglia cells *in vitro* (Figure 3(a)). In this part, the expression of IL-1*α* and C1q that induce the formation of A1 astrocytes in the brain tissue of EAE mice was detected. As showed in Figures 7(a) and 7(b), the level of IL-1*α* and C1q mRNA in EAE group was significantly increased, while APS prominently suppressed the expression of these two factors. These partial results suggest that APS could inhibit the secretion of inflammatory factors inducing the formation of A1 astrocytes in EAE mice. Further, we observed the effect of APS on the expression of A1 astrocyte marker molecules C3 and GFAP. The results showed that APS obviously decreased the expression levels of C3 and GFAP mRNA, as showed in Figures 7(c) and 7(d). It was indicated that APS inhibited the formation of A1 astrocytes. Combined with the previous results, we found that APS could inhibit the polarization of microglia to M1 type and thus suppress the secretion of inflammatory factors that induce A1 astrocytes by M1 type microglia and then obstruct the formation of A1 astrocytes.

## 4. Discussion

In recent years, the effective ingredients of Chinese herbal medicine have attracted the attention of many scholars because of their multichannel and multitarget therapeutic effects. APS is one of the most commonly used in Chinese medicines for the treatment of diseases such as neuroinflammation and neurodegenerative diseases [26]. Preliminary studies in our laboratory have found that the water extract of *Astragalus membranaceus* and its active ingredient APS could effectively treat EAE mice, inhibit neuroinflammation and demyelination, and suppress the proliferation of MOG specific T cells [6]. Furthermore, we demonstrated that APS could promote myelin regeneration through inducing the differentiation of oligodendrocytes from neural stem cells in cuprizone model of demyelination [27]. In addition, we also demonstrated that APS could regulate microglia activation *in vitro* [28]. Based on these, in this study, we explored the regulation of APS on microglia polarization balance in the treatment of EAE and its possible mechanism.

Extensive activation of microglia is a crucial factor in mediating CNS neuroinflammatory damage. Microglia is activated under the condition of stimulation and can be polarized into M1 type or M2 type after activation. In the activation phenotype of microglia, a significant increase in the number of M1 phenotypes brings about the damage of myelin, axons, and neurons; on the contrary, the increase in the number of M2 phenotypes contributes to the alleviation of the disease [29]. Accordingly, regulating the polarization of microglia from the proinflammatory phenotype to the anti-inflammatory phenotype may be of great benefit to treating CNS inflammatory diseases. Related studies have shown that naringenin, an active ingredient in the traditional Chinese medicine orange-red, could regulate the M1/M2 phenotype of microglia through the MAPK signaling pathway and produced antineuritis effects [30]. Another related study showed that Bu Shen Yi Sui Capsule could alleviate neuroinflammation and demyelination by promoting microglia toward M2 polarization by regulating miR-124 and miR-155 in EAE [31]. Therefore, we speculated that the effective treatment of EAE by APS is likely to be through the regulation of microglia M1/M2 polarization phenotype. The study results conformed that APS could inhibit the polarization of microglia to the M1 proinflammatory phenotype *in vivo* and *in vitro* and promote their polarization to the M2 anti-inflammatory phenotype, thereby inhibiting the neuroinflammation mediated by activated microglia. However, we found some differences *in vivo* and *in vitro* experiments. In activated microglia induced by LPS *in vitro*, M1 type increased, and M2 type decreased, and APS intervention reversed this imbalance. In EAE mice, both M1 and M2 type microglia were all increased, mainly because the body upregulated the anti-inflammatory response of M2 type in a strong inflammatory environment. But it was difficult to resist the proinflammatory effect of M1 type, leading to the progression of the disease. APS reduced M1 microglia and further increased M2 microglia, thus reducing CNS inflammation and demyelination.

Although little is known about the mechanism of microglia polarization, some current studies support that the polarization state of microglia can change under certain conditions, and reactive oxygen species such as ROS and miR-155 play a vital role in microglia polarization [32, 33]. Studies have found that the upregulated expression of miR-155 in activated microglia or macrophages can promote the polarization of these cells to a proinflammatory phenotype while blocking miR-155 in microglia can reduce the release of inflammatory mediators and microglia-mediated neurotoxicity [33, 34]. Besides, the expression of miR-155 was also found to increase significantly in the active lesions of multiple sclerosis patients, indicating that the miR-155 pathway not only participates in the regulation of inflammatory response but also promotes the polarization of microglia to M1 type. Therefore, the miR-155 pathway is considered an attractive target for the treatment of autoimmune diseases of the central nervous system. A recent study showed that Bu Shen Yi Sui Capsule promoted the transformation of microglia from M1 type to M2 type by regulating miR-155 and miR-124 in effective treating EAE [31]. Resveratrol could promote the M2 type transformation of microglia by inhibiting the expression of miR-155, thus improving the neuroinflammation after cerebral ischemia [35]. Apigenin reduced the number of M1 type microglia by inhibiting the expression of miR-155 but does not promote the increase of M2 type microglia, in treating retinal degeneration [36]. In this study, we found that APS inhibited the expression of miR-155 in the brain of EAE mice and in the activated microglia induced by LPS. In addition, it was found that APS could also inhibit the expression of miR-155 in microglia stimulated by miR-155 selective agonist *in vitro*. It is important that the selective inhibitor of miR-155 significantly abolished the inhibitory effect of APS on M1 type and obviously canceled the promotion effect of APS on M2 type, which suggested that the regulation of M1/M2 polarization phenotype balance by APS may be mediated by miR-155. However, there are some deficiencies in this part of the study. For example, the blocking experiment *in vivo* was not conducted to confirm the phenotypic regulation of APS on microglia via miR-155 in EAE mice. Meanwhile, we did not further observe the effects of APS on the target genes of the miR-155 pathway. It is not clear whether the effect of miR-155 on microglia phenotypic polarization is direct or indirect. In our subsequent experiments, we will further explore the direct downstream target genes of miR-155 to verify the effect of luciferase reporter gene and more precise targets of APS in regulating microglia phenotypic polarization.

**Figure 8:**
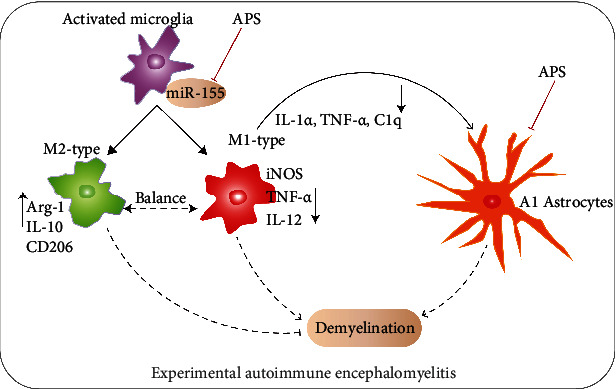
Schematic diagram manifesting that the balance of M1/M2 polarization phenotype in microglia via miR-155 was regulated and the A1 astrocytes were inhibited by APS in EAE. During the development of CNS inflammation and demyelination in EAE, microglia were activated first and polarized into M1 and M2 types. M1 type and M2 type were unbalanced, M1 type increased significantly, M2 type was relatively less. M1 type secreted a large number of proinflammatory factors, which on the one hand induced activation of A1 astrocytes, on the other hand to induce demyelination combined with A1 astrocytes. M2 type plays an anti-inflammatory role to inhibit demyelination. APS treatment inhibited M1 type polarization, promoted M2 type polarization, and restored the equilibrium of M1/M2 of activated microglia via miR-155. APS reduced the secretion of proinflammatory factors by M1, decreased the A1 astrocytes, and promoted the secretion of anti-inflammatory factors by M2 type, which together inhibited demyelination.

Astrocytes of A1 neurotoxic phenotype can secrete a large number of proinflammatory cytokines and neurotoxic factors, which on the one hand can induce the death of oligodendrocytes and neurons and on the other hand can interact with microglia to further expand inflammation. Recently, a relative study has shown that methylprednisolone could decrease microglia activation, suppress A1 astrocytes activation, and promote functional recovery after acute traumatic spinal cord injury mouse models [37]. Another study revealed that estrogen can inhibit the activation of microglia and A1 phenotype neurotoxic astrocytes, significantly reducing neuroinflammatory injury in traumatic brain injury [38]. Liu et al. found that cottonseed oil treatment alleviated ischemic stroke injury by reducing microglia and astrocytic activation and inflammation, which was related to the reduction of A1 phenotype neurotoxic astrocyte activation [39]. Our team's previous studies have confirmed that APS can inhibit CNS inflammation and demyelination in EAE [6]. In this study, it has been confirmed that APS could inhibit microglia from becoming a classic M1 proinflammatory phenotype, reduce the cytokines TNF-*α*, IL-1*α*, and C1q that induce A1 astrocytes formation, and thus suppress the formation of A1 neurotoxic astrocytes, ultimately alleviating demyelination induced by CNS inflammation in EAE mice (Figure 8).

## 5. Conclusions

APS regulates the M1/M2 polarization balance of microglia, inhibits the M1 polarization, and promotes the M2 polarization. miR-155 may be mediated the regulation of APS on microglia polarization balance. APS reduces the secretion of inflammatory factors of activated microglia and suppresses the activation of A1 neurotoxic astrocytes, so as to improve CNS neuroinflammation and demyelination and play a benefit role in the treatment of EAE. This study provides new evidence for APS as an effective drug for the treatment of CNS neuroinflammatory diseases, as well as new pathways of action and therapeutic strategies.

## Figures and Tables

**Table 1:** Primer sequence used in the experiments.

Gene	Primer sequence
TNF-*α*	Forward:5′-ATGTCTCAGCCTCTTCTCATTC-3′
	Reverse:5′-GCTTGTCACTCGAATTTTGAGA-3′
iNOS	Forward:5′-GTT CTC AGC CCA ACA ATA CAA GA-3′
	Reverse:5′-GTG GAC GGG TCG ATG TCA C-3′
IL-12	Forward:5′-AGT GAC ATG TGG AAT GGC GT-3′
	Reverse:5′-CAG TTC AAT GGG CAG GGT CT-3′
Arg-1	Forward:5′-CAT ATC TGC CAA AGA CAT CGT G-3′
	Reverse:5′-GAC ATC AAA GCT CAG GTG AAT C-3′
CD206	Forward:5′-CCT ATG AAA ATT GGG CTT ACG G-3′
	Reverse:5′-CTG ACA AAT CCA GTT GTT GAG G-3′
IL-10	Forward:5′-TTCTTTCAAACAAAGGACCAGC-3′
	Reverse:5′-GCAACCCAAGTAACCCTTAAAG-3′
U6	Forward:5′-CGCTTCGGCAGCACATATAC-3′
	Reverse:5′-AAATATGGAACGCTTCACGA-3′
miR-155	Forward:5′-TGCGCTTAATGCTAATTGTGATA-3′
	Reverse:5′-CCAGTGCAGGGTCCGAGGTATT-3′
IL-1*α*	Forward:5′-CGAAGACTACAGTTCTGCCATT-3′
	Reverse:5′-GACGTTTCAGAGGTTCTCAGAG-3′
C1q	Forward:5′-AAAGGCAATCCAGGCAATATCA-3′
	Reverse:5′-TGGTTCTGGTATGGACTCTCC-3′
C3	Forward:5′-CCAGCTCCCCATTAGCTCTG-3′
	Reverse:5′-GCACTTGCCTCTTTAGGAAGTC-3′
GFAP	Forward:5′-CGGAGACGCATCACCTCTG-3′
	Reverse:5′-AGGGAGTGGAGGAGTCATTCG-3′
GAPDH	Forward:5′-AGGTCGGTGTGAACGGATTTG-3′
	Reverse:5′-TGTAGACCATGTAGTTGAGGTCA-3′

## Data Availability

The result data used to support the findings of this study are included within the article.

## Supplementary Materials

Supplementary Materials To screen the appropriate concentration of APS in BV2 cells, the cell viability experiment by CCK8 assay was conducted. It was found that APS at the dose of 0.8 mg/ml significantly inhibited the viability of BV-2 cells, while APS at the other three doses of 100 *μ*g/ml, 200 *μ*g/ml, and 400 *μ*g/ml did not. Therefore, we chose the maximum one of 400 *μ*g/ml *in vitro* experiments (See Figure S1).
