# Supplementary figures and images for: Microglia Polarization from M1 toward M2 Phenotype Is Promoted by Astragalus Polysaccharides Mediated through Inhibition of miR-155 in Experimental Autoimmune Encephalomyelitis
Source: Oxid Med Cell Longev. 2021 Dec 24;2021:5753452. doi: 10.1155/2021/5753452 (PMC8720009; doi:10.1155/2021/5753452)

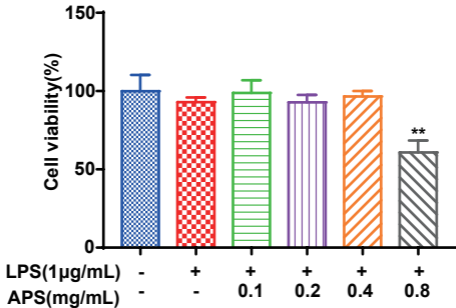

Supplement: Supplementary Materials — To screen the appropriate concentration of APS in BV2 cells, the cell viability experiment by CCK8 assay was conducted. It was found that APS at the dose of 0.8 mg/ml significantly inhibited the viability of BV-2 cells, while APS at the other three doses of 100 μg/ml, 200 μg/ml, and 400 μg/ml did not. Therefore, we chose the maximum one of 400 μg/ml in vitro experiments (See Figure S1). [file 5753452.f1.zip › Figure S1.pdf]
